# Serotonin syndrome and cannabis: A case report

**DOI:** 10.1177/10398562231219858

**Published:** 2023-12-06

**Authors:** Zohaib Nadeem, Chaston Wu, Sophie Burke, Stephen Parker

**Affiliations:** Saint Lucia, QLD; Saint Lucia, QLD; and Chermside, QLD; Saint Lucia, QLD; and Chermside, QLD; Saint Lucia, QLD; and Chermside, QLD

Dear Editor,

The increasing availability of cannabis administration devices (e.g. ‘vapes’ and ‘dab pens’) and medicinal cannabis with high ∆9-tetrahydrocannabinol (THC) concentrations may introduce novel complications of cannabis use. Cannabinoids predominantly act through the endogenous cannabinoid system, but animal studies suggest these compounds can also stimulate serotonin receptors.^1^ This is in addition to CYP450 interactions altering the metabolism of serotonergic agents.^2^ Recent cases from the United States suggest that high concentrate cannabis use can precipitate serotonin syndrome in patients taking antidepressants.^3–5^ We report a case, with the informed consent of the patient, that suggests recurrent induction of serotonin syndrome requiring hospitalisation in the context of cannabis use.

A 20-year-old Asian man presented to the emergency department on three occasions via ambulance within 3 weeks with features consistent with serotonin syndrome, based on the Hunter Serotonin Toxicity Criteria (see Supplementary Table). He had an established diagnosis of bipolar disorder managed with fluoxetine 40 mg, melatonin 10 mg, and lithium (12 h-level ∼0.7 mmol/L).

On Occasion 1, he presented with tonic-clonic seizures following a medication overdose (fluoxetine 560 mg, unknown quantity lithium). UDS was positive for THC. On Occasion 2, he presented with a GCS 10, febrile, tachycardic, hypertensive, restless and agitated. Examination revealed hyperreflexia, symmetrical ankle clonus and intermittent right calf clonus. An unremarkable outpatient assessment the previous day corroborated the acuity of onset. On recovery, he disclosed oral ingestion of cannabis oil (THC 28.5 mg/mL + cannabidiol < 1 mg/mL, unknown quantity) 1–2 hours before the episode. On Occasion 3, he presented as agitated, uncooperative and climbing off the bed. He was diaphoretic, GCS 13, tachycardic and hypertensive, and displayed horizontal jerky nystagmus, and had lower limb clonus and hyperreflexia. Collateral history suggested onset over <3 hours. Upon recovery, he disclosed using a THC ‘vape pen’ < 3 hours before the onset of altered consciousness. We acknowledge the limited information from patient records, including unquantified THC levels.

Cannabis products were present on all occasions where serotonin syndrome emerged. For Occasions 2 and 3, there was clear evidence that cannabis products were used immediately before the onset of symptoms. Given emerging evidence regarding the potential for cannabis to precipitate serotonin syndrome, a cannabis use history should be taken before prescription of serotonergic medications. Patients should be cautioned about the risks of co-administration of cannabis products.

## Supplemental Material

Supplemental Material - Serotonin syndrome and cannabis: A case report Click here for additional data file. Supplemental Material for Serotonin syndrome and cannabis: A case report by Zohaib Nadeem, Chaston Wu, Sophie Burke and Stephen Parker in Australasian Psychiatry
