# Supplementary material for: Serotonin syndrome and cannabis: A case report
Source: Australas Psychiatry. 2023 Dec 6;32(1):100–1. doi: 10.1177/10398562231219858 (PMC10809727; doi:10.1177/10398562231219858)
Supplement: Supplemental Material - Serotonin syndrome and cannabis: A case report [file sj-pdf-1-apy-10.1177_10398562231219858.pdf]

Supplementary Table. Details of each presentation of serotonin syndrome.

		Occasion 1	Occasion 2	Oc[illegible]
Timeframe of occasions		Reference (0-days)	6 days since Occasion 1	18 days si[illegible]
Prescribed medications		Lithium (12-hour level 0.7mmol/L)<br>Fluoxetine 40mg PO MANE [c]<br>Melatonin 10mg PO NOCTE	Lithium (12-hour level 0.7mmol/L)<br>Fluoxetine 40mg PO MANE<br>Melatonin 10mg PO NOCTE	Lithium (12-ho[illegible]<br>Fluoxetine [illegible]<br>Melatonin [illegible]
Medication overdose		Fluoxetine 560mg PO<br>Lithium (unknown quantity)<br>Timeframe before ED presentation is not established (<8 hours)	None [d]	N[illegible]
Cannabis use		Evidenced by positive UDS only	Cannabis oil (THC 28.5mg/mL + cannabidiol<1mg/mL) PO, unknown quantity taken 1-2 hours before ED presentation. Prescription for cannabis oil was dated 9 months earlier.	Inhalation via vap[illegible]<br>(unknown concentra[illegible]<br>pres[illegible]
Absence of serotonin syndrome features at last medical review		Five days prior	4 days prior	2 d[illegible]
Last witnessed in the absence of serotonin syndrome features		Two hours prior	<5-hours prior	<3-h[illegible]
Documented features at ED presentation		Ambulance note: 'GCS 10 (E4, V1, M5) and dilated pupils'.<br>On initial Emergency Department review 'significant diaphoresis whole body, HR 130-140bpm sinus tachy, normotensive… pupils 7mm bilaterally and reactive, patient hypertonic with persistent ankle clonus'.<br>Rapid emergence of tonic-clonic seizures.	Ambulance note: 'Acute behavioural disturbance. Restless… Temp 37.4°C. SR 125, systolic BP 150, BGL 6.3'.<br>On initial Emergency Deparment review: Temp 37.7°C, HR 119, BP 154/95, RR 24, GCS=10 (E4, V1, M5). The following were reported on examination 'restless and agitated', bilateral patellar and ankle hyperreflexia, symmetrical ankle clonus and intermittent right calf clonus.	Ambulance no[illegible]<br>consciousness, leth[illegible]<br>H[illegible]<br>On initial Emerger[illegible]<br>'Diaphoretic, confu[illegible]<br>but responding to v[illegible]<br>able to provide any r[illegible]<br>examination: diap[illegible]<br>(37.5°C), HR135, BP[illegible]<br>nystagmus, pupils 5r[illegible]<br>clonus (>30 beats), a[illegible]
Hunter criteria[a]	Spontaneous clonus	Yes	-	[illegible]
	Inducible clonus and Agitation/diaphoresis	-	Yes	[illegible]
	Ocular clonus and Agitation/diaphoresis	-	-	[illegible]
	Inducible clonus/Ocular clonus and Hypertonia+hyperthermia	-	-	[illegible]
	Tremor and Hyperreflexia	-	-	[illegible]
Severity of serotonin syndrome symptoms [a]		Severe	Moderate	M[illegible]
Timeframe to resolution		~24-hours [e]	<18 hours	<[illegible]
Hospital-based interventions		Intubation > ICU admission, Levitiracetam and midazolam.	Diazepam + IV antibiotics, supportive care, ICU monitoring	Midazolam, Car[illegible]<br>telemetry, Susportiv[illegible]<br>benzo[illegible]

[a] See: Wang HZ, Vashistha V, Kaur S, Houchens NW. Serotonin syndrome: preventing, recognizing, and treating it. Cleveland Clinic Journal of Medicine. 2016;83([illegible]

[b] Severity categorisation based on the classification outlined in Wang HZ, Vashistha V, Kaur S, Houchens NW. Serotonin syndrome: preventing, recognizing, and tre[illegible] Journal of Medicine. 2016;83(11):810-817.

[c] Commenced at 20mg 28 days prior, increased to 40mg 20 days prior.

[d] The patient did not have access to medications at these times (supported administration)

[e] Timeframe to extubation and wakening in ICU
